# The Fly Sensitizing Pigment Enhances UV Spectral Sensitivity While Preventing Polarization-Induced Artifacts

**DOI:** 10.3389/fncel.2018.00034

**Published:** 2018-02-07

**Authors:** Marko Ilić, Andrej Meglič, Marko Kreft, Gregor Belušič

**Affiliations:** Department of Biology, Biotechnical Faculty, University of Ljubljana, Ljubljana, Slovenia

**Keywords:** polarization vision, sensitizing pigment, *Drosophila*, microvilli, rhabdomere, polarimetry

## Abstract

Microvillar photoreceptors are intrinsically capable of detecting the orientation of e-vector of linearly polarized light. They provide most invertebrates with an additional sensory channel to detect important features of their visual environment. However, polarization sensitivity (PS) of photoreceptors may lead to the detection of polarization-induced false colors and intensity contrasts. Most insect photoreceptors are thus adapted to have minimal PS. Flies have twisted rhabdomeres with microvilli rotated along the length of the ommatidia to reduce PS. The additional UV-absorbing sensitizing pigment on their opsin minimizes PS in the ultraviolet. We recorded voltage from *Drosophila* photoreceptors R1–6 to measure the spectral dependence of PS and found that PS in the UV is invariably negligible but can be substantial above 400 nm. Using modeling, we demonstrate that in R1–6 without the sensitizing pigment, PS in the UV (PS*_UV_*) would exceed PS in the visible part of the spectrum (PS*_VIS_*) by a factor PS*_UV_*/PS*_VIS_* = 1.2–1.8, as lower absorption of Rh1 rhodopsin reduces self-screening. We use polarimetric imaging of objects relevant to fly polarization vision to show that their degree of polarization outdoors is highest in the short-wavelength part of the spectrum. Thus, under natural illumination, the sensitizing pigment in R1–6 renders even those cells with high PS in the visible part unsuitable for proper polarization vision. We assume that fly ventral polarization vision can be mediated by R7 alone, with R1–6 serving as an unpolarized reference channel.

## Introduction

Microvillar or rhabdomeric photoreceptors have the structural prerequisite to sense the orientation of e-vector of incident light (De Vries et al., 1953; Snyder and Laughlin, 1975). Each microvillus contains many molecules of the light sensing pigment, rhodopsin. Its chromophore is preferentially aligned along the long axis of the microvillus, so that the photon absorption probability is highest if the e-vector is parallel to the long axis and lowest if it is perpendicular to the long axis; the ratio of probabilities yields the dichroic ratio *Δ* of a microvillus (Snyder and Laughlin, 1975; Goldsmith and Wehner, 1977; Roberts et al., 2011). A fly photoreceptor harbors tens of thousands of microvilli organized into a long, slender light sensing organelle, the rhabdomere. A rhabdomere with perfectly aligned microvilli renders a photoreceptor high polarization sensitivity (PS), which is smaller than Δ due to self-screening, except in the special case of a crustacean-type, interdigitated rhabdom (Snyder, 1973), found also in the horsefly retina (Wunderer et al., 1990). Many insects possess polarization vision by combining photoreceptors with different polarization sensitivities to detect important features in the environment (Horváth, 2014). However, polarization-sensitive photoreceptors absorb a smaller fraction of photons from a non-polarized source than photoreceptors with the same dimensions, but no PS. Further, PS may result in the perception of polarization-induced false colors and intensity contrasts (Wehner and Bernard, 1993; Kelber et al., 2001; Kinoshita et al., 2011). Thus, the PS of the visual channel serving motion or color vision is often minimized by the rotation of the rhabdomere along its longitudinal axis, i.e., the rhabdomeric twist (Smola and Wunderer, 1981a,b; Wehner and Bernard, 1993; Wernet et al., 2012), or additionally, as in the case of the fly neural superposition (Braitenberg, 1967; Kirschfeld, 1967; Agi et al., 2014), by the convergence of R1–6 cells with different PS axes on common interneurons (McCann and Arnett, 1972). The insect retina therefore consists mainly of photoreceptors with minimal PS, while specialized photoreceptors with maximal PS are contained within distinct subpopulations, often localized in special regions, e.g., the dorsal rim area (DRA) (Labhart and Meyer, 1999, 2002). In order to be able to analyze the e-vector orientation, photoreceptors with high PS typically occur as couples with a common field of view and orthogonally crossed rhabdomeres, forming polarization-opponent analyzer pairs (Labhart, 2016; Heras and Laughlin, 2017).

Each ommatidium in the retina of Diptera contains six photoreceptors named as R1–6 and two photoreceptors R7 and R8. Cells R1–6 have six separated rhabdomeres and are used primarily to detect achromatic contrasts and mediate motion vision; cells R7 and R8 share a common rhabdomere R7,8 (R7 distal, R8 proximal) and are used primarily to detect color and polarization (Hardie, 1985; Wernet et al., 2015). The functions of R1–6 and R7,8 partially overlap (Wardill et al., 2012; Schnaitmann et al., 2013). Most R1–6 and R7,8 rhabdomeres are twisted in order to keep their PS minimal (Seifert et al., 1985). Straight R7,8 rhabdomeres with high PS are found in the DRA and in the ventral retina of horseflies (Wunderer and Smola, 1986; Smith and Butler, 1991) and in rather rare cases in fruitflies (Wernet et al., 2012). Those in the DRA detect the polarized sky pattern and help the flies to navigate (Hardie, 1984; Weir et al., 2016), while those in the ventral retina might mediate the polarotactic attraction of horseflies toward linearly polarized reflections from shiny animal fur and water bodies (Horvath et al., 2008). Ideally, the photoreceptors in the opponent pairs should have identical spectral sensitivities, so that the spectral composition of the observed motifs would not influence the polarization-opponent signal. Thus, the R7,8 in the fly DRA express a single, UV-sensitive opsin Rh3 (Fortini and Rubin, 1990). In *Drosophila*, PS in the ventral retina (VPS) is probably mediated by a subpopulation of the ommatidial type named pale (p), which contains broadband or blue-sensitive R1–6, UV-sensitive R7p, and blue-sensitive R8p receptors (Wernet et al., 2012). This receptor combination is clearly not optimized for a spectrally balanced polarization vision. The other subtype is named yellow (y) and contains UV-sensitive R7y and UV-green sensitive R8y (Wernet et al., 2015). Interestingly, VPS remains functional even in *Drosophila* with genetically silenced photoreceptors: only R7p and R1–6 photoreceptors are sufficient for VPS in the UV, and R1–6 are sufficient for VPS in the green. It has been proposed that VPS in the green is mediated by specialized R1–6 with less twisting (low-twist) rhabdomeres (Wernet et al., 2012).

It is important to notice that the blue-sensitive rhodopsin Rh1 molecule of fly R1–6 photoreceptors is fitted with one or two molecules of UV-absorbing sensitizing pigment, 3-hydroxyretinol (Hardie, 1985). The extra chromophore, which resides at the external side of the opsin moiety, can excite the central chromophore via the Förster resonant transfer of energy of an absorbed photon (Kirschfeld et al., 1983). Consequently, a rhodopsin gains sensitivity in the UV, as the β sensitivity peak in the UV is increased from ∼0.25 to possibly > 2 (normalized to the α-peak in the blue or green) (Stavenga, 2004). However, the sensitizing pigment completely suppresses the PS between 300 and 400 nm (Guo, 1981; Vogt and Kirschfeld, 1983), indicating that its molecules are not dichroic or are not aligned with the microvillus. The sensitizing pigment might thus have evolved, in addition to the rhabdomeric twist, as a means to eliminate polarization-induced artifacts, especially if the degree of polarization (DOP) of objects in the natural environment is high in the UV. However, the combination of a UV-sensitive R7p, needed for VPS with a broadband-sensitive R1–6 with low twist, and a sensitizing pigment might not function as a proper polarization-opponent pair. We hypothesize that low-twisting R1–6 cells could be detectable with electrophysiological recordings as photoreceptors that have high PS in the blue–green. Additionally, some of these cells might have reduced sensitizing pigment and restored PS in the UV.

Here, we use microelectrodes to measure the spectral and PS of *Drosophila* photoreceptors R1–6. The spectral dependence of PS is then used to assess the influence of the sensitizing pigment to the PS of R1–6 and R8. Finally, we use polarimetric imaging to estimate the DOP of objects across the spectrum. The linearly polarized reflections are spectrally broad and always have a prominent UV component. We conclude that it is very unlikely that under natural illumination, *Drosophila* R1–6 could analyze polarized visual signals.

## Materials and Methods

### Electrophysiology

Experiments were performed in 2–7 days old, red-eyed *Drosophila* Canton S strain, raised on a standard, corn-based medium. The electroretinogram (ERG) extracellular voltage recordings were made with blunt borosilicate micropipettes, filled with insect ringer. Intracellular recordings were made with sharp quartz micropipettes (resistance *R* = 100–200 MΩ), filled with 3 M KCl. Stimulation was provided with a Xe arc lamp, a monochromator (77250-M, Newport Oriel, United States; bandpass full-width at half-maximum ≈ 10 nm), quartz lenses, and a fused silica optical fiber (1000 μm diameter; Ocean Optics, United States), positioned on a goniometric cardan arm. The tip of the fiber appeared to the fly as a point source with 2° aperture. PS was measured with monochromatic flashes, presented through a rotating polarizer (OUV2500; Knight Optical, United Kingdom). Responses were transformed into sensitivities *S*, which were then fitted with a cos^2^ function

$$S(\alpha )={A\left[\cos\left(\alpha +\phi \right)\right]}^{2}+C$$

where α is the e-vector angle, *A* the amplitude, and *ϕ* the phase shift and *C* the offset. PS was calculated as PS = *S*_max_/*S*_min_; for details, see Belušič et al. (2016, 2017).

### Modeling Polarization Sensitivity

Polarization sensitivity was calculated using a discrete model, modified from Wernet et al. (2012). A rhabdomere was sliced into 1 μm cylindrical segments with uniform microvillar orientation at angle *ϕ_m_*. Rhodopsin absorption spectra were either calculated in 5 nm intervals in the range 300–600 nm_,_ using a rhodopsin template (Stavenga et al., 1993), taken from the published data (R8y; Hardie, 1985) or from own measurements (sensitizing pigment; Belušič et al., 2016). The dichroic ratio *Δ* was either constant, *Δ* = 10, or in the presence of the sensitizing pigment varied as a function of wavelength, *λ*: *Δ_λ_* = 1 (300 < *λ* < 370 nm), 1 < *Δ_λ_ <* 10 (sigmoidally increasing from 1 to 10 for 370 < *λ* < 400 nm), *Δ_λ_* = 10 (*λ* > 400 nm). The incident light was spectrally neutral and non-polarized. Each segment absorbed a fraction of light *κ*, depending on the e-vector angle *ϕ* and wavelength *λ*; the angle-dependence of the absorption coefficient followed the function

$$\kappa \left(\phi ,\lambda \right)=2\kappa \left(\lambda \right)(1+(\Delta -1)\cos^{2}\left(\phi -\phi_{m}\right)){\left(\Delta +1\right)}^{-1}$$

where the absorption coefficient, integrated across the angles and wavelengths, was *κ* = 0.005 μm^-1^. The remaining light was passed to the next segment. The angle-dependent maximum and minimum of the integrated absorption at each wavelength yielded the PS of the photoreceptor as a function of wavelength. To test the effect of R7 as a polarization filter influencing PS of R8, the light exiting R7 was passed into R8; distal R7 and R8 microvilli were mutually orthogonal.

### Polarimetric Imaging

The imaging system was assembled of a monochrome camera (BFLY-PGE-09S2M-CS, FLIR systems, United States), a near-UV achromatic lens (diameter *D* = 12.5 mm, focal distance *f* = 25 mm; #65-971, Edmund Optics, United Kingdom) mounted in a focusing helicoid (SM1NR05, Thorlabs, Germany), bandpass filters with bandwidth 40 nm, center wavelengths 360 nm (with IR blocking coating; Chroma, United Kingdom), 450, 525, 600 nm (Techspec, Edmund Optics, United Kingdom), mounted in a motorized filter wheel (FW102C, Thorlabs, Germany) and a broadband, UV-enabled polarization filter (OUV2500; Knight Optical, United Kingdom), mounted on a motorized rotator (G065118000, Qioptiq, Germany). Image acquisition and filter rotation were controlled with a microcontroller (Uno, Arduino, Italy) and camera software (FlyCapture2, FLIR Systems, United States). Sequences of six images at polarizer angles α = 0–150° in 30° steps were obtained at each bandpass filter. For each pixel, the modulation of intensity (*I*) was extracted as a function of α and the sequence was fitted with a cos^2^ function (Eq. 1; sensitivity substituted with intensity).

The *DOP* was calculated from the function maximum and minimum (*I*_max_, *I*_min_) as

$$DOP=\frac{I_{\max}-I_{\min}}{I_{\max}+I_{\min}}$$

All analyses were done using Matlab (MathWorks, United States). Objects were imaged outdoors in Ljubljana, Slovenia, on clear summer days around noon, or indoors, illuminated with a Xe arc lamp.

## Results

**FIGURE 1:**
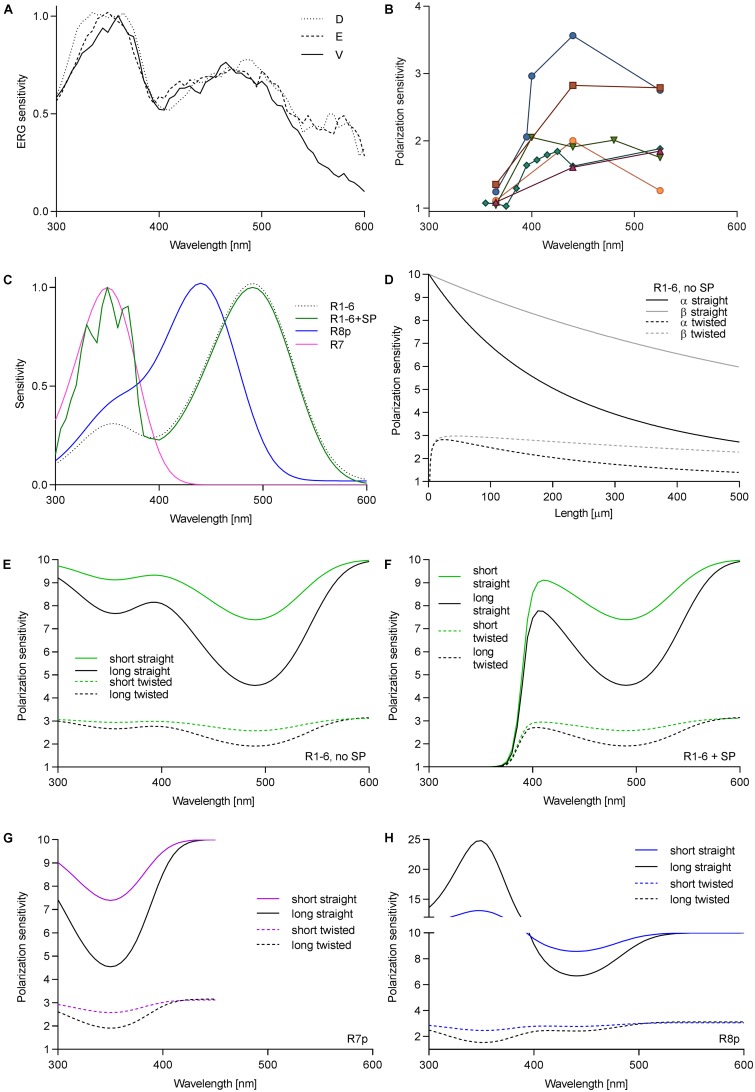
Wavelength dependence of PS in fly photoreceptors. **(A)** Spectral sensitivity of *Drosophila*, measured via ERG in dorsal (D), equatorial (E), and ventral (V) retina. **(B)** PS of single *Drosophila* R1–6 cells as a function of wavelength; different colors represent individual cells (*N* = 6). **(C)** Spectral sensitivities of fly photoreceptors; R1–6, Rh1 (*λ*_max_ = 486 nm), R7p, Rh4 (*λ*_max_ = 355 nm); R8p, Rh5 (*λ*_max_ = 440 nm). **(D)** PS of R1–6 without sensitizing pigment as a function of rhabdomere length and twist in the blue (PS_α_) and in the UV (PS_β_). Theoretical PS, calculated in short rhabdomeres (*l* = 80 μm), long rhabdomeres (*l* = 250 μm), with straightly aligned or twisting microvilli, in R1–6 without sensitizing pigment **(E)**, with sensitizing pigment **(F)**, R7p **(G)**, and R8p **(H)**.

We first measured the spectral sensitivity of *Drosophila* to check whether the sensitizing pigment was reduced or absent in R1–6 in the ventral retina. Spectral sensitivity measurements via ERG in three regions (dorsal, equatorial, and ventral) revealed a large sensitivity peak in the UV and a smaller peak in the blue. The ratio between the peaks *S*_UV_/*S*_B_ = 1.37 did not vary among the regions (**Figure 1A**). Measurement in single cells yielded *S*_UV_/*S*_B_ = 1.74 ± 0.46 (mean ± SD, *N* = 6). Thus, the variation of the ratio of rhodopsin Rh1 to sensitizing pigment is the same in the various eye regions. We further measured PS of single cells in the ventral retina as a function of wavelength. PS was consistently low in the UV (PS = 1.04–1.35), but it was moderate and quite variable in the visible part (PS = 1.6–3.6; **Figure 1B**).

To evaluate the effect of the sensitizing pigment on PS of the different photoreceptors in pale ommatidia (spectral sensitivities in **Figure 1C**, case of R1–6 presented with or without sensitizing pigment), we calculated the PS of long (*Calliphora*, 250 μm) and short (*Drosophila*, 80 μm) rhabdomeres (occupancy of length by cell type: R1–6 100%, R7 60%, R8 40%; R7 and R8 perpendicular to each other), straightly aligned or progressively twisted along the length by 90°. The effect of self-screening in a straight and twisting R1–6 without the sensitizing pigment on the PS at the rhodopsin α peak (480 nm) and its β peak (350 nm) is presented in **Figure 1D**. PS in the UV is always higher than in the blue and remains substantial even in a long and twisted rhabdomere (PS_UV_/PS_V IS_ = 1.2–1.8; **Figures 1D,E**). The wavelength dependence of PS in R1–6, R7p, and R8p is presented in **Figures 1E–H**. The sensitizing pigment minimizes PS in the UV in R1–6 to ∼1 (**Figure 1F**) Filtering by R7p (**Figure 1G**) can increase PS of R8p with a straight rhabdomere in the UV high above the value imposed by the dichroic ratio *Δ* = 10 (maximum PS = 25; **Figure 1H**). The case of R7y (UV-sensitive receptor with a sensitizing pigment) is trivial (always PS = 1; not shown); however, the underlying R8y which has a green opsin and a sensitizing pigment (Hardie and Kirschfeld, 1983) does not gain PS by the filtering in R7y, as the distal photoreceptor does not act as a polarizer (Hardie et al., 1979).

**FIGURE 2:**
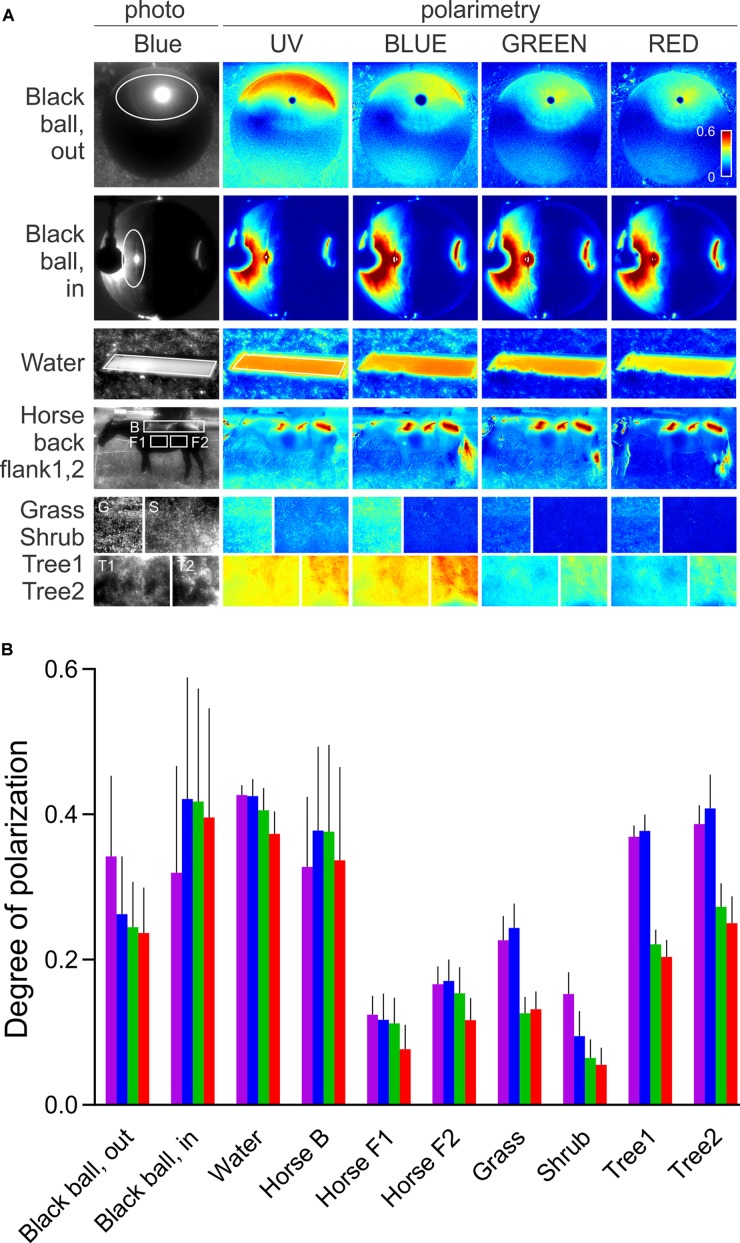
Spectral dependence of DOP of a horsefly trap (black shiny ball), water surface, fur of a black horse, and vegetation. **(A)** Polarimetric images (columns 2–5); DOP in UV, blue, green, and red, presented with false colors; regions of interest are marked with ellipses and rectangles in the non-polarimetric photograph in the blue channel (column 1). **(B)** DOP of regions of interest in **(A,B)**. Colors of bars correspond to the spectral bands. Error bars correspond to standard deviation.

We have shown that the sensitizing pigment can minimize PS in the UV, at the Rh1 β-peak. To evaluate the ecophysiological importance of polarization in the UV, we measured the DOP of objects, relevant to the visual ecology of flies. Very little is known about fruitfly polarization vision, and the horseflies represent the only family of Diptera where polarotactic behavior has been demonstrated convincingly. Thus, we have imaged horsefly attractants (a shiny black beach ball, a horse), and polarized objects with broader significance for insect visual ecology (water surface, vegetation). The images were obtained in four spectral bands: UV, blue, green, and red. In polarimetric images (**Figure 2A**), we designed regions of interest with high DOP (indicated in the non-polarimetric photo in the blue channel) and calculated the probability density functions (PDFs) of DOP. The spectral dependence of DOP, plotted as the mean and SD of the PDF for each spectral band, is presented in **Figure 2B**. Under any circumstances, the DOP of reflections is substantial in all parts of the spectrum. In objects illuminated with skylight (black ball outdoors, trees, horse’s flank), DOP is the highest in the UV or blue and lower at longer wavelengths. In objects illuminated with direct sunlight (horse’s back) or an artificial light source (black ball, indoors), DOP does not depend on the wavelength, or is slightly lower in the UV and red. The angle of polarization (not shown) is approximately horizontal (water, horse, vegetation) or varies circularly (black ball). The movements of horse’s tail and whisperer resulted in small DOP artifacts, out of the region of interest.

## Discussion

Our results show that in *Drosophila* R1–6, PS is moderate and variable in the blue and green parts of the spectrum, corresponding to the various degrees of rhabdomeric twist, as demonstrated in serial anatomical sections (Wernet et al., 2012). However, PS of R1–6 is always very low in the UV, rendering R1–6 useless as the opponent analyzer in the pair with R7, although the rare putative cases of R1–6 with high PS in the UV might have been missed in our recordings. Furthermore, the reflections in the nature are always spectrally broad and highly polarized in the UV. Thus, due to the sensitizing pigment, even those R1–6 with high PS in the blue cannot function as an opponent channel to R8 while observing outdoor reflections. As the DOP of the horsefly attractants is highest in the UV and blue, it seems likely that the horsefly ventral polarization vision is optimized by employing the UV (and blue) sensitive central photoreceptors, without the sensitizing pigment. Here, a significant role might be attributed to R8p that gains very high PS in the UV by the aid of filtering in R7p and consequently remains polarization-sensitive even with a twisted rhabdomere. The importance of the UV channel in fly polarization vision is demonstrated by transgenic *Drosophila* which appear to align their bodies with ventrally presented polarized light using the combination of UV-sensitive R7p and broadband photoreceptors R1–6 (Wernet et al., 2012). It appears that this could be achieved during locomotion by comparing the fluctuating signal from R7p with a stable signal from the polarization-insensitive channel, R1–6. Anyhow, this combination of photoreceptors cannot properly analyze the e-vector and may lead the insect to confuse the DOP with light contrast (Labhart, 2016). Interestingly, we have recently identified a similar single-channel polarization detection system in the ventral retina of moths (Belušič et al., 2017). Similar to R7p and R8p, moth PS photoreceptors are maximally sensitive to blue and UV, hence adapted to the spectral maximum of DOP of objects in nature. Polarized reflections of skylight and sunlight are not wavelength-neutral, even in objects that reflect spectrally neutral polarized light under artificial illumination (**Figure 2**). The reflections are spectrally altered by the optical properties of the illuminant and of the reflector. Illumination by the sun results in spectrally neutral reflections, while the scattered and partially polarized light from the sky results in a UV and blue maximum of DOP. Generally, an object may produce specular and diffuse reflections. DOP is inversely proportional to the relative amount of scattered light and is high in those parts of the spectrum, where the absorbance of an object is high. Chlorophyll and melanin strongly absorb in the UV and blue and hence result in a higher DOP at short wavelengths and in lower DOP in the green part of the spectrum. UV and blue peaking PS photoreceptors (i.e., fly R7,8 pale) seem to be optimal for detecting polarized reflections. On the other hand, the sensitizing pigment appears as a very efficient unpolarizing agent, as the DOP of reflections is high in the UV. In fly motion vision pathway, signals from R1–6 with differently oriented rhabdomeres converge on a common laminar neuron, so that their PS is canceled out (McCann and Arnett, 1972). In the case of R1–6, the sensitizing pigment primarily acts to increase photon catch in the UV and thus the signal-to-noise ratio. In R7,8 (i.e., in UV-sensitive R7y and green-sensitive R8y), the sensitizing pigment improves receptor’s resilience to polarization-induced spurious contrasts.

## Author Contributions

MI performed the electrophysiological experiments, modeling, and data analysis and programed the polarimetric camera. AM performed the polarimetric imaging and analyzed the images. MK developed the image analysis software. GB initiated and supervised the study, assembled the figures, and wrote the manuscript. All authors have contributed to the final version of the manuscript.

## Conflict of Interest Statement

The authors declare that the research was conducted in the absence of any commercial or financial relationships that could be construed as a potential conflict of interest. The reviewer FL and handling Editor declared their shared affiliation.
